# Supplementary material for: Risk factors for sacrococcygeal pilonidal sinus: a systematic review and meta-analysis supplemented by genetic causal assessment
Source: Front Surg. 2026 Jan 7;12:1718589. doi: 10.3389/fsurg.2025.1718589 (PMC12819706; doi:10.3389/fsurg.2025.1718589)
Supplement: Supplementary file 2 [file Datasheet2.zip › Supplementary Data 2/MR_pipeline_p5e-6/leaveone_plots/ebi-a-GCST005814.finngen_R12_L12_PILONIDALCYST.pdf]

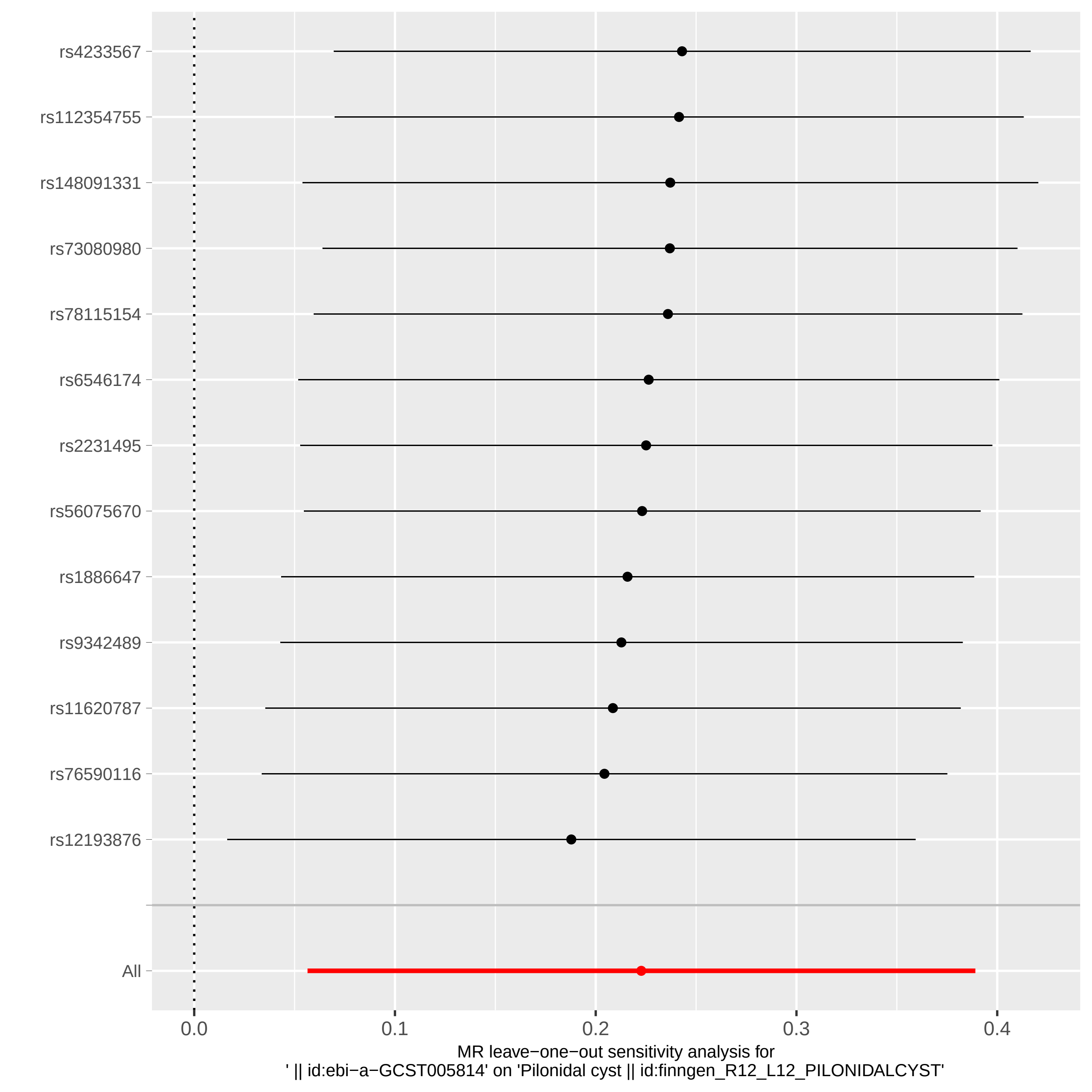
rs4233567
rs112354755
rs148091331
rs73080980
rs78115154
rs6546174
rs2231495
rs56075670
rs1886647
rs9342489
rs11620787
rs76590116
rs12193876
All
0.0
0.1
0.2
0.3
0.4
MR leave−one−out sensitivity analysis for
' || id:ebi−a−GCST005814' on 'Pilonidal cyst || id:finngen_R12_L12_PILONIDALCYST'
